# A New Approach to an Old Concept for Reducing Shoulder Pain Caused by Gynecological Laparoscopy

**Published:** 2018

**Authors:** Shahla Chaichian, Bahram Moazzami, Ameneh Haghgoo, Kourosh Sheibani

**Affiliations:** 1- Minimally Invasive Techniques Research Center in Women, Tehran Medical Sciences Branch, Islamic Azad University, Tehran, Iran; 2- Pars Advanced and Minimally Invasive Medical Manners Research Center, Pars Hospital, Iran University of Medical Sciences, Tehran, Iran; 3- Basir Research Center, Tehran, Iran

**Keywords:** Gynecology, Iran, Laparoscopy, Pain, Shoulder, Surgery

## Abstract

**Background:**

The purpose of this study was to introduce a technique to extract the remaining peritoneal gas in order to improve the post-laparoscopic shoulder pain.

**Methods:**

This study included 12 patients undergoing laparoscopic gynecologic procedures between February and March 2016 in Minimally Invasive Techniques Research Center, Pars Hospital, Tehran, Iran. For complete suction of the air from abdominal cavity, the air was first vacuumed from the pelvic cavity in Trendelenburg position and then the patients were put in anti-Trendelenburg position. In this position, as the remaining gas was shifting toward subdiaphragmatic area, the suction tube was shifted to a position next to the camera canal and the remaining air was suctioned. A 10 point visual analogue scale was used to measure the severity of patients’ post-operative shoulder pain.

**Results:**

The mean VAS for shoulder pain was 0.8±1.7 4 *hr* post-surgery. At 12 *hr* post-surgery, the mean VAS was 0.8±1.5. At 24 *hr* post-surgery, the mean VAS for shoulder pain was 0.3±0.8. Finally, 48 *hr* post-surgery, the VAS score for all patients was zero.

**Conclusion:**

Our approach for emptying the abdominal cavity from residual gas after laparoscopic procedures seems to be useful in preventing post-operative shoulder pain among patients undergoing gynecological laparoscopic surgeries. Further studies are suggested to compare the effect of our proposed method with other methods.

## Introduction

Pain management after laparoscopic procedures has an important impact on patients’ satisfaction (1, 2). Abdominal, shoulder, and back pain occurs frequently after laparoscopic gynecological procedures (2). The etiology of the post-laparoscopic pain is very complex (2). This pain is usually felt in abdomen and also shoulder areas (3). The suggested sources of abdominal pain include disruption of tissues, and port site pain (1, 4). Previous studies have indicated 35% to 80% incidence of shoulder pain following laparoscopy (3, 4). Carbon dioxide (CO_2_) is considered to be the main cause of post-operative shoulder pain attributed to its peritoneal stretching and irritation of the diaphragm. Several studies have shown a relation between the residual gas volume and the severity of post-operative shoulder pain (3–7).

To reduce the post-laparoscopic shoulder pain, several methods have been suggested including the use of a peritoneal gas drain in the first 4–6 hours following laparoscopy, intraperitoneal local anaesthesia, pulmonary recruitment maneuver, intraperitoneal saline infusion, gasless laparoscopy and reduction in insufflation pressure (8–14). It has been suggested that these methods reduce post-operative shoulder pain by decreasing the volume of residual intraperitoneal gas (3, 15, 16), but there is no consensus among researchers regarding the effectiveness of above mentioned methods (17–20).

In the present study, a technique to extract the remaining abdominal gas among patients who undergo uncomplicated laparoscopic gynecologic procedures was evaluated regarding its effectiveness in reducing the post-laparoscopic shoulder pain.

## Methods

The Minimally Invasive Techniques Research Center, Pars hospital ethics committee approved this case series study and informed written consent for conducting and later publication of the study results was obtained from all participants before the start of study. The study was conducted in Minimally Invasive Techniques Research Center, Pars hospital, Tehran, Iran, from February to March 2016 and included twelve patients with age range of from 17 to 59 years who underwent uncomplicated gynecological laparoscopic procedures. Patients with any history of pre-operative shoulder pain were excluded from the study. Also, patients with a history of pulmonary disorder, abdominal or pelvic pain, tubo-ovarian abscess and severe adhesions in abdominal or pelvic regions were excluded from the study. All patients entering the study were operated by the same surgical team. All patients were given intravenous Cefazolin (1 *g*) following induction of the general anesthesia as a prophylactic antibiotic. Laparoscopy was performed by direct trocarization and CO_2_ insufflation. Two 5 *mm* ports were inserted through outer upper margins of bilateral rectus muscle sheaths, a 10 *mm* port through umbilicus for telescope insertion, and then a suprapubic trocar as described before (21). CO_2_ was insufflated with the rate of 2 *l/min* for the first step of procedure and the gas pressure was maintained in 12–16 *mmHg* range throughout the procedure as described before (21). Post-operative pain was controlled using NSAIDs. If the patients complained of pain post-operatively, oral doses of 100 *mg* diclofenac were prescribed. These findings were recorded for all participants: age, body mass index (BMI), nausea and/or vomiting, and the time to first gas passing. Also, the duration of laparoscopic procedure, post-operative hospital stay and any complications were recorded as well. A 10 point visual analogue scale (VAS) was used to measure the severity of patients’ post-operative shoulder pain at 4, 12, 24 and 48 *hr* post-operatively. The score 0 in VAS scale indicated “no pain” and the score 10 indicated the worst pain possible and patients were asked to give a score from 0 to 10 based on their severity of pain. The pain score was assessed by nursing staff, who were blinded regarding the aims and design of the study. The main objectives of the present study were to evaluate shoulder pain at 4, 12, 24 and 48 *hr* after the surgery and the demand for analgesic medications.

### Technique for gas extraction:

Complete suction of the air from abdominal cavity after laparoscopy was performed as follows: The air from the pelvic cavity in Trendelenburg position was vacuumed and then the patients were put in anti Trendelenburg position. In this position, as the remaining gas was shifting toward subdiaphragmatic area, the suction tube was shifted to a position next to the camera canal and the remaining air was suctioned with reducing speed mode to prevent any shift of internal organs toward the suction pores. The process was stopped after complete air evacuation under optimal vision. At the end of the surgery, the suction and umbilical trocar were discharged under careful visual examination.

## Results

All 12 patients entering the study completed the laparoscopic surgery and the follow up period. Table 1 shows the patients baseline characteristics. The mean age of patients was 33 years and the most common cause of the laparoscopy was endometriosis resection (4 patients) followed by the resection of other ovarian cysts.

**Table 1.** Patients’ baseline characteristics

**Variable**	**Mean±SD**	**Median (range)**
**Age**	33.3±12.6	32 (17 to 59)
**BMI**	23±3.2	23 (16.2 to 28.2)
**Bleeding (*ml*)**	383±159	400 (200 to 600)
**Type of surgery**		**n(%)**
Endometriosis resection		2 (16.7%)
Endometriosis resection +DIE		1 (8.3%)
Myectomy		2 (16.7%)
Partial hysterectomy		1 (8.3%)
Resection of other ovarian cysts		3 (25.0%)
Resection of other ovarian cysts +Myectomy		1 (8.3%)
Total hysterectomy		2 (16.7%)

At 4 *hr* post-surgery, the mean VAS for shoulder pain was 0.8±1.7 and VAS reading of higher than 2 indicating a considerable pain was detected in 1 patient. At 12 *hr* after surgery, the mean VAS was 0.8±1.5 and considerable shoulder pain was observed in 1 patient. At 24 *hr* after surgery, the mean for shoulder pain was 0.3±0.8 and none of patients had considerable shoulder pain. Finally, 48 *hr* post-surgery, the VAS score for all patients was zero (Table 2).

**Table 2.** Shoulder and abdominal pain after surgery

**Time**	**Shoulder**	**Abdomen (VAS)**	**Abdomen (Touch)**
**4 *hr***			
Mean±SD	0.8±1.7	1.8±2.1	3.3±2.3
Median (range)	0 (0 to 6)	1.5 (0 to 8)	2 (1 to 9)
**12 *hr***			
Mean±SD	0.8±1.5	2.6±2.6	3.8±2.6
Median (range)	0 (0 to 5)	2 (0 to 9)	3 (1 to 10)
**24 *hr***			
Mean±SD	0.3±0.8	1.6±1.7	2.3±2.2
Median (range)	0 (0 to 2)	1 (0 to 6)	2 (0 to 8)
**48 *hr***			
Mean±SD	0.1±0.3	0.7±1	1.1±1.2
Median (range)	0 (0 to 1)	0 (0 to 3)	1 (0 to 4)

The mean VAS score for abdominal pain at 4, 12, 24 and 48 *hr* after surgery measured by VAS and abdominal touch is summarized in table 2. The mean number for analgesics demand in the first day after surgery, the second day after surgery and thereafter is summarized in table 3. The mean duration of hospitalization for our patients was 2.1±1.2 days; with only one patient staying at hospital for more than 48 *hr* (Table 3). Table 3 also shows the time for food tolerance, gas passing and urination after the surgery as well as surgical complications. There was only one patient (8.3%) with surgical complication (partial bowel resection due to endometriosis adhesion).

**Table 3.** Analgesics demand, the time for food tolerance, gas passing, urination after the surgery as well as the number of surgical complications and the length of hospital stay among patients

**Variable**	**Mean±SD**	**Median (range)**
**Analgesics**		
First day	1.4±0.9	1.5 (0 to 3)
Second day	0.9±1	1 (0 to 3)
After the second day	0.5±0.7	0 (0 to 2)
Total	2.8±2.2	2.5 (0 to 7)
**Food tolerance**	6±3.6	5.5 (1 to 15)
**Urination**	8.8±3.9	9 (4 to 18)
**Gas passing**	3.3±1	3 (2 to 6)
**Duration of hospital stay (Days)**	2.1±1.2	--

## Discussion

Abdominal and shoulder pain are common after laparoscopic surgery. Retained carbon dioxide (CO_2_) in abdominal cavity is considered to be the main cause of post-operative shoulder pain and some researchers have indicated a relation between the residual gas volume and the severity of post-operative shoulder pain (3–7, 21). Different methods including drainage have been used to remove the gas in the peritoneal cavity and reduce the post-operative pain with different degrees of reported effectiveness (21–24).

In the present study, it was found that our approach for complete removal of residual gas after uncomplicated gynecological laparoscopic procedures has an acceptable effect on shoulder pain. No drain was placed after using our method which potentially simplifies the post-operative patient care and potentially shortens the length of hospital stay.

The mean VAS score for shoulder pain after using our method was around 0.8±1.7 at the first 4 post surgical hours and it was reduced to zero after 48 *hr*. In comparison, Kerimoglu et al. (4) have reported a VAS average of 4.1 at six *hr* and 0.8 at twenty-four *hr* after laparoscopy when there was no drain implementation and 2.7 and 0.9, respectively when they put a drain in place. An average abdominal pain of 1.8±2.1 four *hr* after laparoscopy was found which is somehow lower than Kerimoglu et al.’s (4) findings who have reported a mean VAS score of 4.1 and 2.7 for abdominal pain 6 and 12 *hr* after laparoscopy without drain placement and a mean score of 5.3 and 2.9, respectively if a drain was kept in place after the surgery. At 12 *hr* after the surgery, our patients had a mean abdominal pain of 2.6±2.6 which was comparable to Kerimoglu et al.’s (4) findings who reported a mean VAS score of 2.7 for abdominal pain without drain placement and a mean score 2.9 if a drain was kept in place at twelve hours after the surgery. Also, our average length of hospital stay was 2.1±1.2 days which is comparable to 1.8 days reported by the same authors (4).

There was only one patient with complication (partial bowel resection due to rectal involvement with endometriosis) and none of patients needed repeating of surgery.

A main limitation of many previous studies examining the effectiveness of drainage in reducing post-laparoscopic shoulder pain is the inclusion of patients with a wide range of surgical indications (21). Our study had a similar limitation since patients undergoing laparoscopy due to different gynecologic disorders were included. The present study also had some other limitations, such as not having a control group and a relatively small sample size.

## Conclusion

Our approach for emptying the abdominal cavity from residual gas after gynecologic laparoscopic procedures seems to be useful in preventing post-operative shoulder pain and also reducing the duration of post-laparoscopic hospital stay. It is probable that this method could be used as a non-invasive technique for reducing post-laparoscopic shoulder pain. Further prospective studies are suggested to compare the effect of our proposed method with previous methods of pain reduction with or without drain placement to confirm its usefulness.
